# Atomic-Resolution Structures of the APC/C Subunits Apc4 and the Apc5 N-Terminal Domain

**DOI:** 10.1016/j.jmb.2015.08.023

**Published:** 2015-10-09

**Authors:** Nora B. Cronin, Jing Yang, Ziguo Zhang, Kiran Kulkarni, Leifu Chang, Hiroyuki Yamano, David Barford

**Affiliations:** 1 Division of Structural Biology, Institute of Cancer Research, 237 Fulham Road, London SW3 6JB, United Kingdom; 2 MRC Laboratory of Molecular Biology, Francis Crick Avenue, Cambridge CB2 0QH, United Kingdom; 3 Division of Biochemical Sciences, Council of Scientific and Industrial Research National Chemical Laboratory, Pune 411008, India; 4 Cancer Institute, University College London, Paul O'Gorman Building, 72 Huntley Street, London WC1E 6BT, United Kingdom

**Keywords:** APC/C, anaphase-promoting complex/cyclosome, cryo-EM, cryo-electron microscopy, EM, electron microscopy, CDK, cyclin-dependent kinase, TPR, tetratricopeptide repeat, SAXS, small-angle X-ray scattering, EDTA, ethylenediaminetetraacetic acid, PEG, polyethylene glycol, Bistris, 2-[bis(2-hydroxyethyl)amino]-2-(hydroxymethyl)propane-1,3-diol, anaphase-promoting complex, ubiquitin, cell cycle, multisubunit structure, protein crystallography

## Abstract

Many essential biological processes are mediated by complex molecular machines comprising multiple subunits. Knowledge on the architecture of individual subunits and their positions within the overall multimeric complex is key to understanding the molecular mechanisms of macromolecular assemblies. The anaphase-promoting complex/cyclosome (APC/C) is a large multisubunit complex that regulates cell cycle progression by ubiquitinating cell cycle proteins for proteolysis by the proteasome. The holo-complex is composed of 15 different proteins that assemble to generate a complex of 20 subunits. Here, we describe the crystal structures of Apc4 and the N-terminal domain of Apc5 (Apc5^N^). Apc4 comprises a WD40 domain split by a long α-helical domain, whereas Apc5^N^ has an α-helical fold. In a separate study, we had fitted these atomic models to a 3.6-Å-resolution cryo-electron microscopy map of the APC/C. We describe how, in the context of the APC/C, regions of Apc4 disordered in the crystal assume order through contacts to Apc5, whereas Apc5^N^ shows small conformational changes relative to its crystal structure. We discuss the complementary approaches of high-resolution electron microscopy and protein crystallography to the structure determination of subunits of multimeric complexes.

## Highlights

• The paper addresses the structure of two large subunits of the anaphase-promoting complex/cyclosome (APC/C): Apc4 and the N-terminus of Apc5, for which no atomic models were previously available.
• Apc4 comprises a WD40 domain split by a long α-helical domain, whereas Apc5^N^ has an α-helical fold.
• Apc4 and Apc5 form a stable heterodimer, presumably an assembly intermediate of the APC/C.
• Based on a cryo-electron microscopy reconstruction of the APC/C, we show how Apc4 and Apc5 interact in the context of the multimeric APC/C complex and highlight the role of evolutionarily conserved surfaces of Apc4 and Apc5 in mediating protein–protein interactions.

## Graphical abstract

**Figure:**
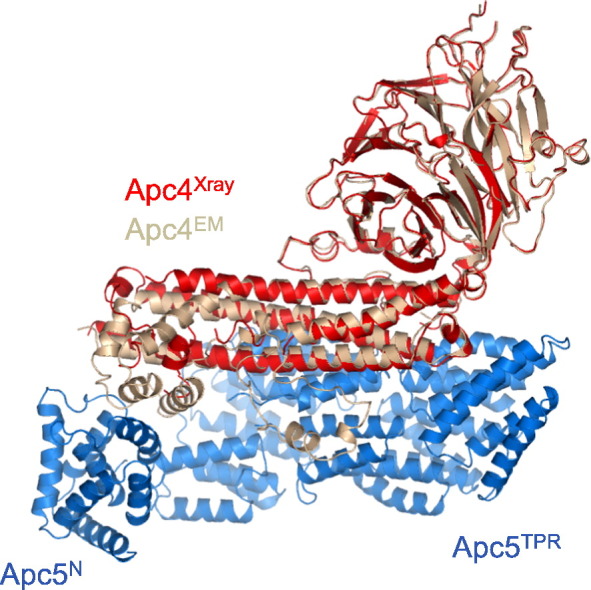

## Introduction

The anaphase-promoting complex/cyclosome (APC/C) is a large multimeric cullin-RING E3 ubiquitin ligase that functions to regulate cell cycle transitions by ubiquitinating specific cell cycle regulatory proteins and targeting them for proteolysis by the ubiquitin-proteasome system [1–5]. In vertebrates, the holoenzyme is a complex of 15 different proteins that assemble into a complex of 20 subunits with a molecular mass approaching 1.2 MDa. Its activity is strictly dependent on the association with the core APC/C of a coactivator subunit (either Cdc20 or Cdh1) that acts both to recruit substrates to the APC/C and to stimulate its E3 ligase catalytic activity [6–15]. Substrate recognition is conferred by short destruction motifs (D box, KEN box and ABBA motifs [16–19]) that interact with coactivator subunits [7–12]. The core APC/C subunit Apc10 contributes to D box recognition through a D box coreceptor formed with coactivator [13,20,21]. Coactivators increase APC/C activity by promoting an increase in affinity for E2 and by enhancing E2 catalytic efficiency [13–15]. Switching coactivators in late mitosis changes substrate specificities and confers the capacity of the APC/C to ubiquitinate substrates in a cell-cycle-dependent manner.

The key role of APC/C^Cdc20^ is to regulate chromosome segregation in mitosis (reviewed in Refs. [2–5]). APC/C^Cdc20^ controls the proteolysis of securin and cyclin B, inhibitors of the protease separase (reviewed in Refs. [1–5]). Activated separase cleaves cohesin, the protein assembly responsible for sister chromatid cohesion. APC/C^Cdh1^ in contrast regulates mitotic exit, the events of cytokinesis and initiation of DNA replication. The APC/C itself controls the switching of Cdc20 and Cdh1 coactivators by regulating the level of cyclin-dependent kinase (CDK)-dependent phosphorylation of core APC/C subunits and Cdh1. Phosphorylation of the APC/C stimulates Cdc20 binding [22,23], whereas Cdh1 phosphorylation negatively regulates its binding to the APC/C [24,25].

A striking feature of the APC/C is its large size. Only four subunits are directly involved in catalyzing protein ubiquitination (the cullin subunit Apc2 and the RING subunit Apc11) [15,26–31] and degron recognition [coactivator subunits (either Cdc20 or Cdh1) and Apc10] [7–13,20,21,32] (reviewed in Refs. [2] and [4]). The remaining 85% of the APC/C mass corresponds to scaffolding subunits, of which seven are large multiple repeat motif proteins (Apc1 and Apc3–Apc8). Four of these are the canonical tetratricopeptide repeat (TPR) proteins Apc3, Apc6, Apc7 and Apc8 that form structurally related homodimers [33–37], whereas Apc1 comprises a proteasome cyclosome repeat domain related in structure to the proteasome cyclosome domain of the proteosomal subunits Rpn1 and Rpn2 [38,39]. Apc5 also includes 13 predicted TPR motifs [40], whereas Apc4 comprises a putative WD40 domain. In addition to coordinating the juxtaposition of the catalytic and substrate recognition modules, scaffolding subunits contribute to catalysis, substrate recognition and regulation. For example, Apc1 and Apc3 incorporate the major CDK-dependent phosphorylation sites [23,41,42] responsible for stimulating Cdc20 association, and in the case of Apc3, these provide docking sites for the Cks subunit of cyclin–CDK–Cks complexes [43].

Through the combination of single-particle cryo-electron microscopy (cryo-EM) of both endogenous and reconstituted complexes with crystallographic analysis and atomic modeling of individual subunits, pseudo-atomic structures of a large proportion of the APC/C have been established. Recently, we reported a 7.4-Å-resolution reconstruction of a ternary complex of human APC/C with the coactivator Cdh1 and a fragment of the high-affinity substrate Hsl1 [13]. This allowed the complete secondary structure assignment of the APC/C and the assignment of all 20 subunits of the complex to their respective segments of the electron microscopy (EM) density map. However, atomic models for Apc4 and the N-termini of both Apc1 (~ 1100 residues) and Apc5 [~ 200 residues (Apc5^N^)] were lacking because no crystal structures were available and their homology to proteins of known structure was insufficient to allow the generation of reliable atomic models. Seeking to obtain insights into these structures at atomic resolution and to assist in the interpretation of cryo-EM reconstructions of the APC/C complex, we determined the crystal structures of full-length Apc4 and the N-terminal domain of Apc5 (Apc5^N^). We docked these atomic models into a 3.6-Å-resolution cryo-EM reconstruction of the human APC/C^Cdh1.Emi1^ complex we had simultaneously determined [44]. Here, we provide a detailed analysis of the crystal structures of Apc4 and Apc5^N^ and the structures of Apc4 and Apc5 within the context of the whole APC/C. In the complex, regions of Apc4 disordered in the crystal assume order through contacts to Apc5, whereas Apc5^N^ shows small conformational changes relative to its crystal structure. The Apc4 WD40 domain (Apc4^WD40^) is well resolved in the crystal structures but is less well defined in the cryo-EM density map due to its peripheral location in the APC/C. We compare and contrast the differences between the crystallographic and EM densities and highlight the complementary approaches of high-resolution EM and protein crystallography to the structure determination of subunits of multimeric complexes.

## Results and Discussion

### Apc4 is a WD40 repeat protein with an α-helical bundle insert

We first determined the crystal structure of *Xenopus laevis* Apc4 (residues 1–741 that lacked the C-terminal 48 residues that were predicted to be disordered [45]). The protein was overexpressed in the insect cell/baculovirus system and the structure was determined using phases to 3.2 Å resolution derived from multiwavelength anomalous diffraction. Tracing of the polypeptide chain was guided by selenomethionine anomalous signals (Supplementary Table 1). We used the *Xenopus* Apc4 coordinates to determine the full-length human Apc4 structure to a similar resolution by molecular replacement (Supplementary Table 2). As a result of contacts with Apc5, regions of Apc4, disordered in both the *Xenopus* and human crystal structures, are defined in the EM density map of the 3.6-Å-resolution cryo-EM reconstruction of human APC/C^Cdh1.Emi1^ [44] (Supplementary Fig. 1).

**Fig. 1:**
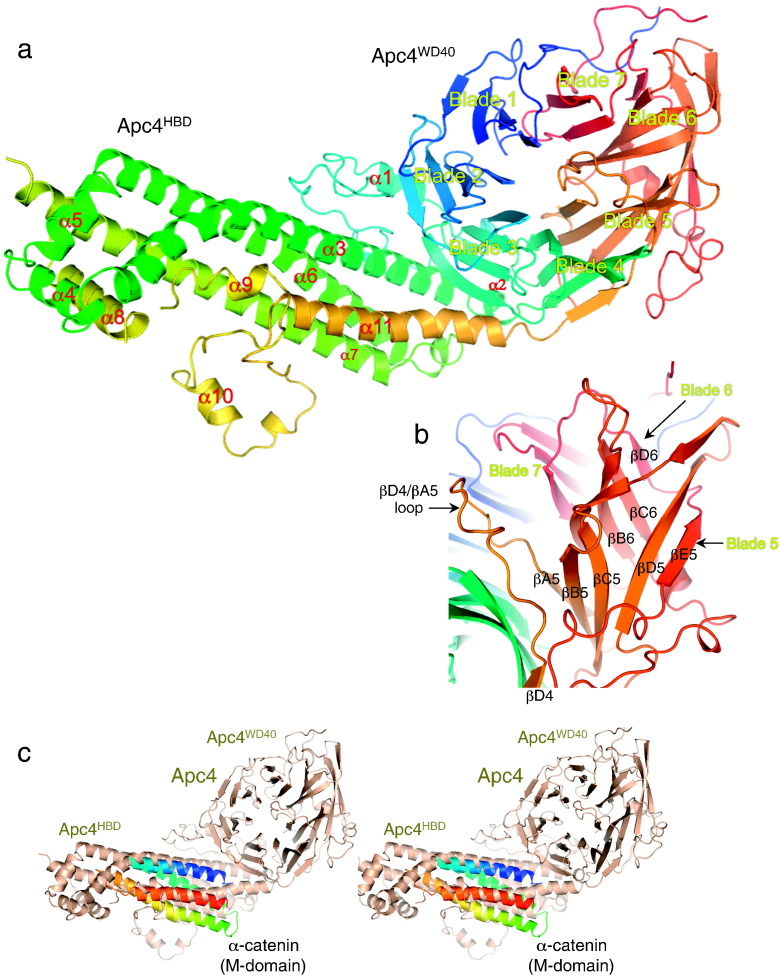
Apc4 comprises a WD40 β-propeller toroid split by a helical bundle domain. (a) Cartoon of Apc4 color-ramped from blue to red from N- to C-termini. Shown is the EM structure of human Apc4. (b) Close-up view of the extended blade 5 of Apc4^WD40^ and showing the βD4/βA5 loop that blocks access to the mouth of the WD40 domain tunnel. (c) Stereoview showing that the M-domain of α-catenin, superimposed onto the EM structure of human Apc4^HBD^, shares structural similarity with the four-helical-bundle domain of Apc4.

The human Apc4 crystal structure was fitted into the 3.6-Å-resolution cryo-EM density map of APC/C^Cdh1.Emi1^ [44] using Chimera [46] (Fig. 1 and Supplementary Fig. 2c). Residues of the Apc4 helical bundle domain HBD (Apc4^HBD^) were well resolved in EM density, and segments of the Apc4^HBD^ not visible in the crystal structures could be built *ab initio* into the EM density, allowing an almost complete atomic model to be generated and refined [44] (Supplementary Fig. 1). C-terminal residues (758–808) of human Apc4 are disordered in both the crystal structure and the EM density map. In contrast, as discussed below, the WD40 β-propeller domain, which is well resolved and ordered in the crystal structures, is less well defined in the EM density, being located at the periphery of the complex (Supplementary Fig. 2b).

Apc4 adopts a bi-domain architecture, dominated by a 360-residue seven-bladed WD40 β-propeller domain (Apc4^WD40^) split by the HBD (Apc4^HBD^) predominantly composed of four long α-helices (Fig. 1a and Supplementary Fig. 1). Apc4^HBD^ is inserted between strands C and D of blade 4 of the WD40 domain, extending away from the wider lower surface of the β-propeller such that, together, the HBD and WD40 domain generate an L-shaped molecule. A second smaller insert between β-strands A and B within blade 3 of Apc4^WD40^ contacts Apc4^HBD^. Finally, an insert between β-strands C and D of blade 6 forms an edge β-strand (βE5) with β-strand D of blade 5 (Fig. 1b and Supplementary Fig. 1). Apart from these three insertions, Apc4^WD40^ resembles a canonical β-propeller architecture as judged by its similarity with other WD40 domain proteins (Table 1) (high DALI *Z* scores [47]). Structure prediction programs were unable to correctly model the β-propeller due to the HBD insert. To our knowledge, the insertion of a helical bundle domain within a WD40 β-propeller domain is novel. The four long α-helices that dominate Apc4^HBD^ share a structural similarity and antiparallel arrangement with the four-helix bundle of domain 2 of the M-fragment of α-catenin [48] (Fig. 1c and Table 1).

**Fig. 2:**
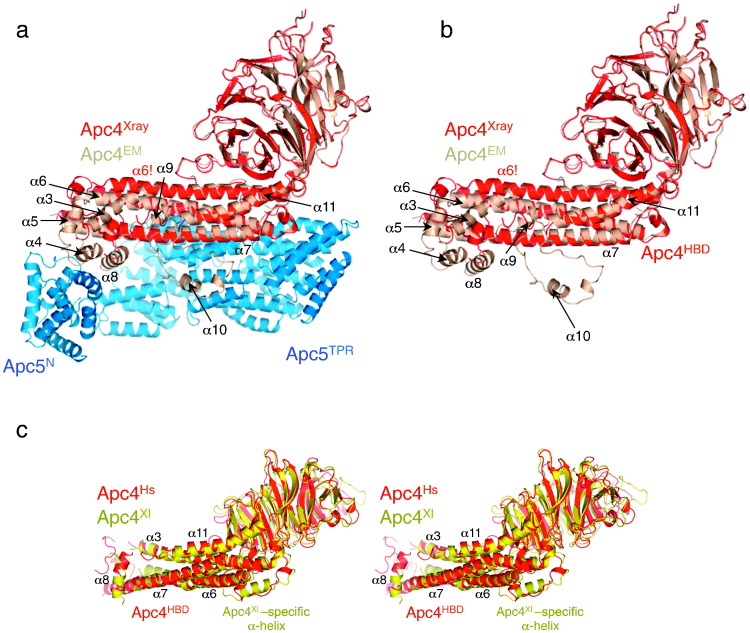
The Apc4–Apc5 protein interface orders regions of Apc4^HBD^. (a) Human Apc4–Apc5 as organized in the APC/C with the X-ray structure of Apc4 (red) superimposed onto the EM coordinates (brown). The Apc4–Apc5 interface is shown. (b) As in (a) but without Apc5. (c) Stereoview showing that the crystal structures of *X*. *laevis* and human Apc4 are very similar (RMSD is 2.1 Å).

There are extensive contacts between Apc4^HBD^ and both the bottom surface and the strand surface of the second, third and fourth blades of Apc4^WD40^ (Fig. 1a). This packing interaction confers stability to the tertiary structure of Apc4, and small-angle X-ray scattering (SAXS) analysis confirmed that the solution structure is consistent with the X-ray structure (Supplementary Fig. 2c). Moreover, superimposing the Apc4 model from the crystal structure onto the EM-derived structure [44] showed that, except for the ordering of regions of Apc4^HBD^, the conformation of Apc4 does not change in the context of the APC/C, indicating that the HBD and WD40 domains are rigidly associated (Fig. 2a and b). Human and *X*. *laevis* Apc4 are also very similar, differing only for residues 120–130 (*X*. *laevis* Apc4 numbering) within the shorter WD40 insert that is an α-helix in the *X*. *laevis* Apc4 structure in contrast to a predominantly disordered loop structure in human Apc4 (Fig. 2c and Table 2).

### Conformational changes of Apc4 from the crystal structures in the context of the whole APC/C

Overall, the crystal and EM structures of Apc4 differ by an RMSD of 2.5 Å (Fig. 2a and Table 2). Differences between the Apc4 crystal and EM structures relate primarily to the tip of Apc4^HBD^. These involve conformational changes and creation of new α-helices. In the context of the APC/C, due to interactions with Apc5, regions of Apc4 that are disordered in the crystal structures adopt ordered α-helical conformations (α4, α9 and α10) (Fig. 2a and b). Helix α4, located at the end of Apc4^HBD^, forms specific contacts to Apc5^N^, whereas α10 becomes ordered due to packing against the TPR domain of Apc5^TPR^. Ordering of the tip of Apc4^HBD^ induces a 45° bend toward the C-terminus of α3, a smaller bend of α6, a shift and extension of α5 and repositioning of α8. Finally, α9 forms to connect α8 with α10.

**Fig. 3:**
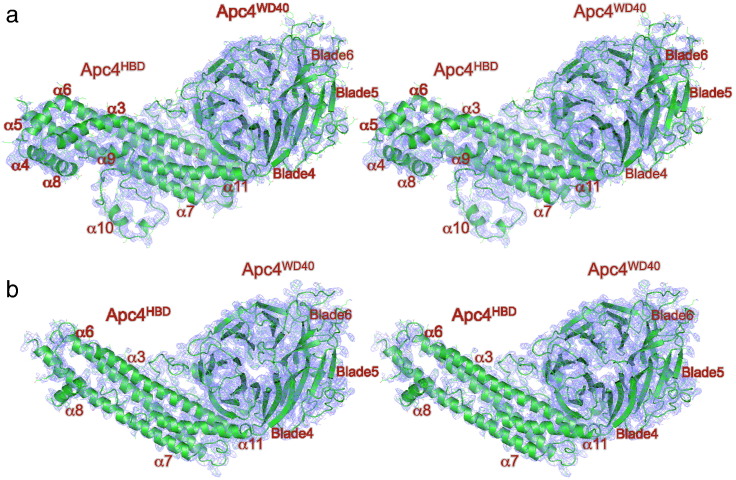
Comparison of EM density maps of APC/C^Cdh1.Emi1^ and crystal structure 2*F*_o_ − *F*_c_ maps of human Apc4. (a) Stereoview of the EM density map and Apc4 coordinates. Main chain is shown as a cartoon. (b) Stereoview of the 2*F*_o_ − *F*_c_ density map contoured at 1σ and Apc4 coordinates. Main chain is shown as a cartoon.

**Fig. 4:**
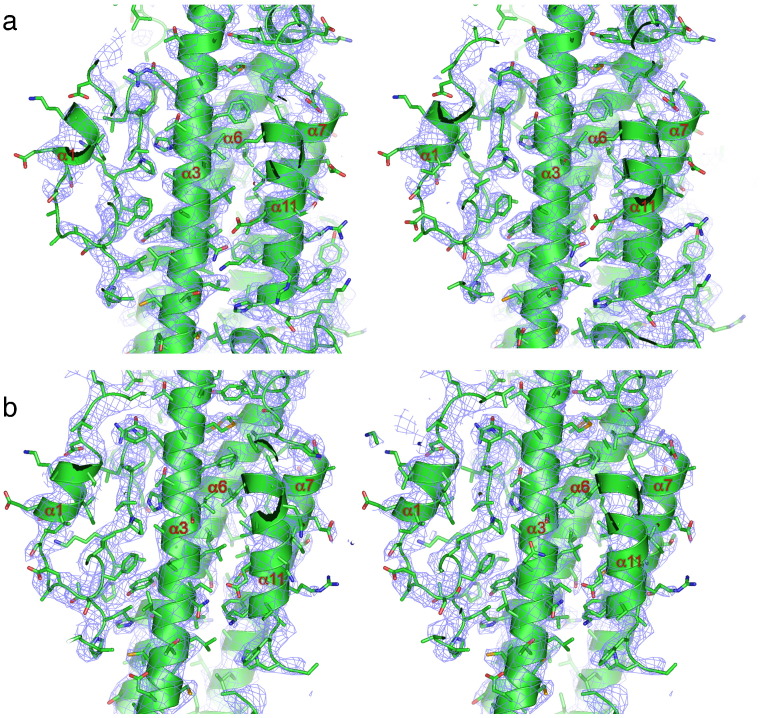
Comparison of EM density maps of APC/C^Cdh1.Emi1^ and crystal structure 2*F*_o_ − *F*_c_ maps of human Apc4^HBD^. (a) Stereoview of the EM density map and Apc4 coordinates. Main chain is shown as a cartoon, and amino acid side chains are shown as sticks. (b) Stereoview of the 2*F*_o_ − *F*_c_ density map contoured at 1σ and Apc4 coordinates. Main chain is shown as a cartoon, and amino acid side chains are shown as sticks.

Figure 3 shows a comparison of the EM density and X-ray (2*F*_o_ − *F*_c_) maps of human Apc4, determined to similar resolutions, 3.6 Å and 3.4 Å, respectively. In the EM density map, secondary structural features are well defined, whereas secondary structural elements at the tip and periphery of Apc4^HBD^ that are resolved in the EM density map are disordered in the X-ray structure (α4, α9 and α10). For both EM and X-ray maps, within the core of Apc4^HBD^, amino acid side-chain densities are of comparable quality, allowing *ab initio* fitting of the polypeptide sequence (Fig. 4).

**Fig. 5:**
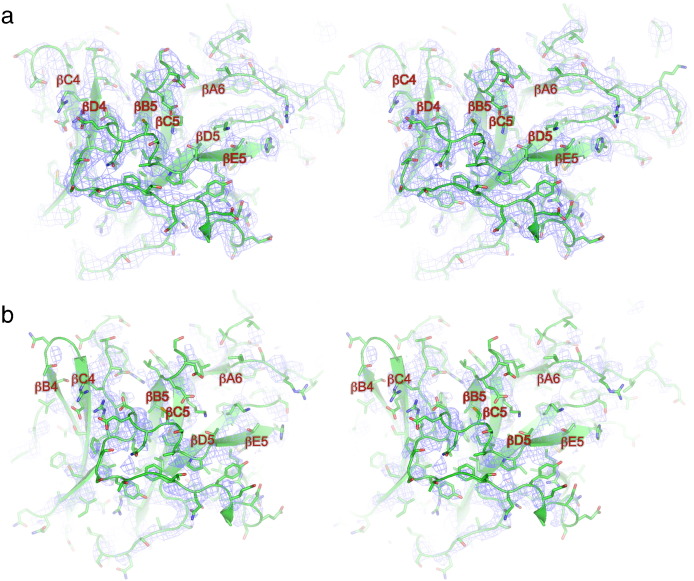
Comparison of EM density maps of APC/C^Cdh1.Emi1^ and crystal structure 2*F*_o_ − *F*_c_ maps of human Apc4^WD40^. (a) Stereoview of the 2*F*_o_ − *F*_c_ density map contoured at 1σ and Apc4 coordinates. Main chain is shown as a cartoon, and amino acid side chains are shown as sticks. (b) Stereoview of the EM density map and Apc4 coordinates. Main chain is shown as a cartoon, and amino acid side chains are shown as sticks.

In contrast, whereas the X-ray map shows well-resolved side-chain densities for residues of Apc4^WD40^ (Fig. 5a), in the EM map, these side chains are poorly defined, particularly for residues of Apc4^WD40^ at the periphery of the APC/C, for example, blades 5 and 6 (Fig. 5b). Thus, regions of Apc4 that assume order only in the context of their interaction with the neighboring Apc5 subunit of the APC/C are visible in the EM map. Apc4^WD40^ is less well defined in the EM map due to its location at the periphery of the molecule where it engages in few intersubunit contacts (Supplementary Fig. 2b), and EM maps have the largest alignment inaccuracies. Additionally, structural variability due to relative intersubunit rotations may be amplified at the periphery of the molecule. In the human Apc4 crystal, Apc4^WD40^ is secured through lattice contacts with two neighboring molecules (data not shown).

### Apc5 is a TPR protein with an N-terminal α-helical domain

Bioinformatics analysis of Apc5 had previously identified Apc5 as a variant TPR protein comprising 13 contiguous TPR-like motifs (residues 206–740), C-terminal to a small α-helical domain [40]. Whereas the canonical TPR is a 34-amino-acid motif [49,50], in Apc5, the repeat varies in length from 34 to 40 amino acids. However, consistent with a TPR protein, the consensus TPR residues (small aliphatic at positions 8, 20 and 27; larger aliphatic residues at positions 1, 4, 7, 10, 24 and 28) [49] are present in Apc5 TPR motifs [40]. Residues 1–208 N-terminal to the TPR domain do not conform to a TPR consensus sequence and no repeat motif was detected using TPRpred [51] and HHrep [52]. Based on secondary structure prediction (Phyre2 [53]) and structure disorder programs (OnD-CRF [45]), residues 1–160 were predicted to be ordered and α-helical, with a disordered linker of ~ 30 residues connecting this helical domain to the TPR domain. We purified residues 1–161 of *X*. *laevis* Apc5 as a bi-product of the Apc4–Apc5^N^ coexpression system and crystallized the protein. The structure was determined to 2.2 Å resolution using single-wavelength anomalous diffraction phasing (Supplementary Table 2).

**Fig. 6:**
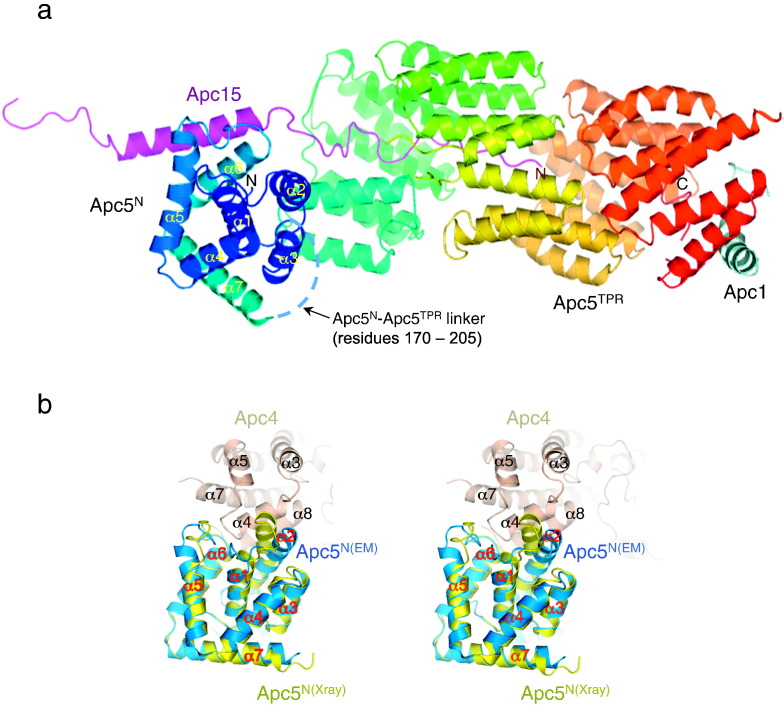
Apc5 has an N-terminal helical domain (Apc5^N^) connected by a disordered linker to a TPR superhelix of 13 TPR motifs (Apc5^TPR^). (a) Cartoon of EM structure of human Apc5 color-ramped from blue to red from N- to C-termini. The small subunit Apc15 that contacts Apc5 is also shown. (b) Stereoview of a superimposition of Apc5^N^ based on human Apc5^N^ EM coordinates (blue) and *Xenopus* Apc5^N^ X-ray (yellow) coordinates. Major structural differences involve the α1/α2 and α5/α6 loops that contact Apc4.

Apc5^N^ [residues 27–161 (human Apc5 numbering)] adopts an all α-helical domain architecture comprising seven α-helices (Fig. 6a and Supplementary Fig. 6). Helices α1–α6 pack to create an antiparallel bundle with α1 at the center of the domain almost completely surrounded by helices α2–α6. This α-helical domain is capped by α7, orientated orthogonally to the α-helical domain. The X-ray structure of *X*. *laevis* Apc5^N^ was readily docked into the assigned density of the 3.6-Å-resolution EM density map of human APC/C^Cdh1.Emi1^ [44] (Supplementary Fig. 2a). A small shift of the loop connecting α5 with α6 accommodates the Apc4–Apc5 interface within the context of the APC/C (Fig. 6b). A larger 6-Å-conformational change involving the loop connecting α1 with α2 and the N-terminus of α2 results from both packing at the Apc4–Apc5 interface and a two-residue insertion within human Apc5 relative to *X*. *laevis* Apc5 (Supplementary Fig. 3). Residues 1–26 of Apc5^N^, predicted to form two α-helices, are disordered in both the crystal structure and the EM density map. No EM density is visible for the loop (residues 170–205) connecting Apc5^N^ with Apc5^TPR^, and thus, as predicted, this loop is disordered. In the crystal structure, residues 164–168 from the TEV cleavage site continue the α7 helix, similar in structure to α7 of human Apc5^N^ defined in the EM density map. DALI searches [47] revealed weak structural similarity to the RNP assembly factor (Table 1), although the biological significance of this similarity is unclear.

Supplementary Figure 4 shows a comparison of the EM density and X-ray (2*F*_o_ − *F*_c_) maps of human Apc5 and *Xenopus* Apc5^N^. The maps determined to resolutions of 3.6 Å and 2.2 Å, respectively, show clear differences in structural detail, although side chains are well resolved in both maps. Trp29 of Apc5^N^ adopts different rotamer conformations in the two structures. The “outward”-facing conformer observed in the crystal structure is hindered in the context of the APC/C due to the close proximity of Apc5^TPR^.

**Fig. 7:**
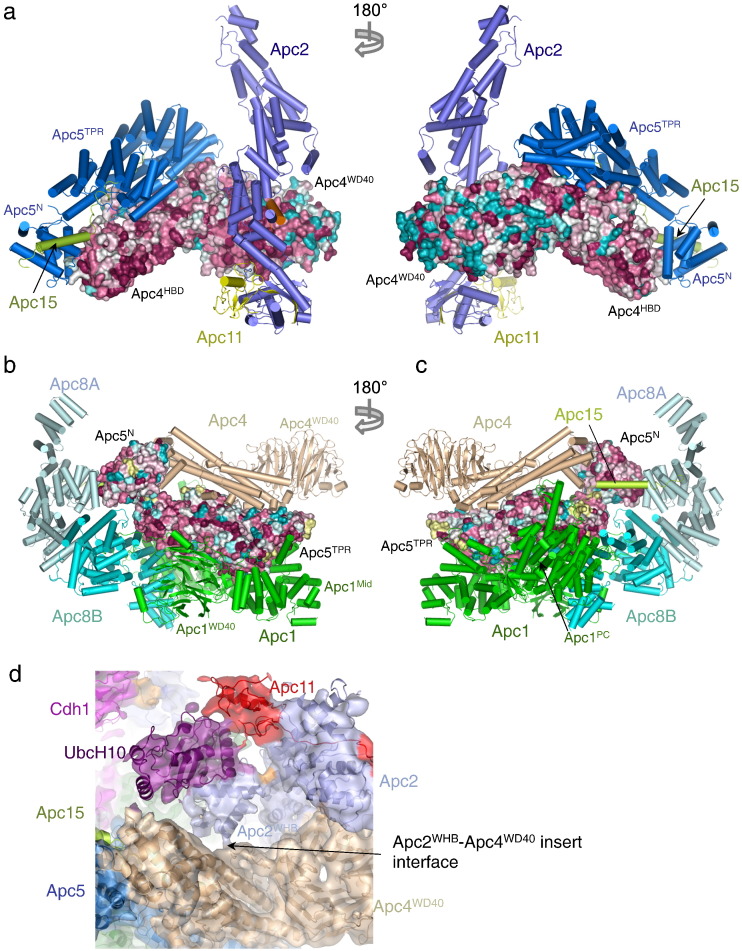
Apc4 and Apc5 interact with neighboring subunits through evolutionarily conserved protein–protein interfaces. (a) Surface of Apc4 color-coded according to conservation (purple, conserved; cyan, unconserved). Contacting subunits Apc2, Apc5 and Apc15 are shown as cartoons. (b and c) Surface of Apc5 color-coded according to conservation (purple, conserved; cyan, unconserved). Contacting subunits Apc1, Apc4, Apc8 and Apc15 are shown as cartoons. (d) Cryo-EM density map of the APC/C^Cdh1.Hsl1^–UbcH10 complex, color-coded according to subunit assignments [44]. Density connects Apc2^WHB^ with the blade 3 insert of Apc4^WD40^, suggesting a direct interaction between these domains. EM coordinates are shown.

A model for Apc5^TPR^ was derived using the TPR superhelix of *Schizosaccharomyces pombe* Apc6 comprising 14 contiguous TPR motifs [35] as a template using Phyre2 [53]. The 3.6-Å-resolution EM density map of human APC/C^Cdh1.Emi1^ was of sufficient quality to fit extended TPR α-helices and model residue side-chain conformations (Supplementary Fig. 2a), resulting in a refined atomic model [44] (Fig. 6a). Except for some instances of longer TPR α-helices, Apc5^TPR^ conforms well to a canonical TPR superhelix, adopting a similar pitch of seven TPR motifs per TPR superhelical turn [49]. Apc5^TPR^ matches most closely with the MALT regulatory protein (DALI *Z* score of 23) (Table 1 and Supplementary Fig. 5a). The TPR superhelices of Apc5 and Apc6 are also similar (DALI score of 16) (Table 1 and Supplementary Fig. 5b). The N-terminus of the TPR superhelix is stabilized by the extended N-terminus of the TPR-accessory subunit Apc15 that lines the internal groove of the TPR superhelix (Fig. 6a), reminiscent of Apc12 interactions with the C-terminal superhelical turn of Apc6 [33,35]. The C-terminal 13 residues of Apc5 fold back into the TPR superhelix, and this segment of Apc5, together with the N-terminus of Apc15, encloses a cavity within the C-terminus of Apc5^TPR^. A 24-residue segment of Apc1 caps the C-terminus of Apc5^TPR^ (Fig. 6a). Apc5^N^ and Apc5^TPR^ domains do not interact directly, and the overall structure of Apc5 is dependent on its extensive interface with Apc4 (Figs. 2a and 7a and b). The absence of direct interactions between Apc5^N^ and Apc5^TPR^ likely accommodates the conformational change within the APC/C platform that is associated with Cdh1 binding and the stimulation of APC/C activity [13].

### Apc4 and Apc5 interact through Apc4^HBD^ and Apc5^TPR^

Apc4 and Apc5 form extensive and stable interactions, burying 6226 Å^2^ (Figs. 2a and 7a and b and Table 3). A heterodimer of Apc4 and Apc5 is isolated as a bi-product of the reconstitution of the apo-APC/C (yeast and human) [40,54] and the two proteins can be stably coexpressed (this work and Ref. [40]). The isolation of an Apc4–Apc5^N^ complex indicates that Apc5^N^ itself interacts with Apc4, consistent with the APC/C structure [44]. However, the tendency of this complex to dissociate during purification indicates that Apc5^TPR^ is required to maintain stable Apc4–Apc5 interactions. At the Apc4–Apc5 interface, the Apc5^TPR^ superhelix packs colinear with Apc4^HBD^, with Apc5^N^ packing against the tip of Apc4^HBD^ (Figs. 2a and 7a and b). The similar size of Apc4^HBD^ and Apc5^TPR^ results in a complementary interface between the two proteins. Conserved surfaces of both subunits comprise the Apc4–Apc5 intersubunit interface (Fig. 7a and b). A stable Apc4 and Apc5 heterodimer suggests the possibility that it exists as an intermediate subcomplex on the APC/C assembly pathway.

### Apc4 and Apc5 interactions with other APC/C subunits

Apc4^WD40^ contacts the N-terminal cullin repeat domain of Apc2. The interaction involves a conserved edge of the β-toroid of Apc4^WD40^ (Fig. 7a and Supplementary Fig. 2b and Table 3). Common interacting surfaces of WD40 domains are entry mouths to the central tunnel. In Apc4^WD40^ of APC/C^Cdh1.Emi1^, these are exposed [44] (Fig. 7a–c and Supplementary Fig. 2b) and that on the lower surface of the domain is 45 Å in diameter, typical of seven-bladed β-propeller proteins. However, in Apc4^WD40^, a tunnel through the β-toroid is blocked by the interstrand loop that connects βD4 and βA5 (Fig. 1b).

Complexes of APC/C^Cdh1^ with UbcH10 (the initiating E2 for human APC/C) showed that contacts to the backside of UbcH10 rigidify the WHB domain of Apc2 (Apc2^WHB^) [30]. This interaction enhances both APC/C–UbcH10 affinity and UbcH10 catalytic activity [30]. Examination of the APC/C^Cdh1.Hsl1^–UbcH10 cryo-EM map [44] revealed EM density linking Apc2^WHB^ with Apc4^WD40^, specifically the small insertion associated with blade 3 (Fig. 7d). This interaction may reinforce the position of Apc2^WHB^, facilitating contacts to UbcH10, thereby contributing to the substantially increased affinity of UbcH10 for the APC/C relative to an isolated Apc2–Apc11 heterodimer, even though UbcH10 only contacts Apc2 and Apc11 [30,44].

In addition to its interface with Apc4, Apc5 forms further extensive interfaces with Apc1, Apc8 and Apc15 (Fig. 7 and Supplementary Fig. 2b and Table 3). All three domains of Apc1 (Apc1^WD40^, Apc1^Mid^ and Apc1^PC^) interact with Apc5^TPR^. The most substantial contacts are contributed by Apc1^WD40^ and Apc1^Mid^, with the edge of Apc1^WD40^ inserting into the groove of the Apc5 TPR superhelix (Fig. 7b). The Apc8 homodimer bridges Apc5^N^ and Apc5^TPR^ (Fig. 7b and c). As described above, the N-terminal segment of Apc15 (residues 1–25) inserts into the superhelix of Apc5^TPR^, whereas residues 26–46 of Apc15 form an α-helix that bridges Apc5^N^ with Apc8A (Fig. 7c).

### Cryo-EM and X-ray crystallography provide complementary information on multiprotein complex structures

A comparison of crystal structures of individual subunits with their conformations in the context of multimeric complexes allows an analysis of the extent of conformational changes resulting from multiple intersubunit interactions. For the APC/C, regions of Apc4 that are disordered in isolation become ordered and stabilized as a result of intersubunit contacts with Apc5 (Figs. 2a and 3). However, the side chains of Apc4^WD40^, which are well defined in the crystal structure, are less well defined in the cryo-EM map (Fig. 5).

To obtain insights into how the other APC/C subunits become ordered through interactions with neighboring subunits in the context of the APC/C, we tabulated regions of disorder of APC/C subunits as observed in crystal structures for comparison with disordered regions of the APC/C (Table 4). This analysis is complicated by the fact that crystallizable proteins often have disordered regions, either defined as being proteolytically sensitive or predicted to be disordered, removed prior to crystallization. However, where data are available, with the exceptions of the C-terminal segments of Apc10 and Cdh1 (discussed below) and Apc4 noted above, there is a good correlation between disordered regions of APC/C subunits observed in crystal structures and in the APC/C, for example, the C-terminal 50–60 residues of Apc3, Apc4 and Apc12, and the N-terminal 26 residues of Apc5. In contrast to their crystal structures, the C-terminal ~ 20 residues of Apc10 and Cdh1 are ordered in the APC/C due to the interaction of their C-terminal IR (Ile-Arg) motifs with the IR tail-binding sites of Apc3 [44]. This suggests that, for the subunits analyzed, protein–protein interfaces are mediated primarily through globular domains. However, we note that the small intrinsically disordered proteins Apc13 and Apc16 (for which there are no or little crystallographic or NMR data) adopt nonglobular structures and mediate numerous intersubunit interactions in the context of the APC/C. Similarly, specific insertions predicted as disordered within the large globular subunits Apc1 and Apc2, for which no crystal structures exist, also mediate intersubunit interactions.

We have noted that, due to its position at the periphery of the complex, the EM density of Apc4^WD40^ is less well defined than that for more ordered centrally located subunits (Supplementary Figs. 2b and 6) [44]. Thus, interpretation of the Apc4^WD40^ cryo-EM density of APC/C^Cdh1.Emi1^ was guided by the Apc4 crystal structure. Some other peripherally located domains and subunits of the APC/C are also defined at resolutions sufficient for definition of secondary structures (e.g., at 5–7 Å) but lower than that required for unambiguous side-chain definition [44] (Supplementary Fig. 6). Interpretation of the EM densities corresponding to these regions of the APC/C relied on existing atomic models from crystal structures and homology modeling. These include the WD40 domain of Cdh1 (Cdh1^WD40^) [18], the catalytic module of Apc2^CTD^ [55] and Apc11 [31] and Apc7 [56]. The catalytic module and Cdh1^WD40^ participate in few contacts to neighboring subunits, and thus, their intrinsic flexibility results in structural variability with concomitant lower resolution in EM density maps. This study illustrates the complementarity of X-ray crystallography and EM approaches to the structure determination of multimeric complexes.

## Materials and Methods

### Apc4 and Apc5^N^ cloning, expression, purification and crystallization

#### Overview of methods

Expression and purification of the human APC/C complex (tagged on Apc4) [54] yielded together with APC/C free Apc4 as identified by mass spectrometry. Initial crystals of Apc4 were obtained using the PACT crystallization screen, which were then optimized. Expression of Apc4 together with Apc5 and Apc15 from human, *X*. *laevis*, *Saccharomyces cerevisiae* and *S*. *pombe* was performed. An Apc4–Apc5 complex was expressed and purified from all four species. However, attempts to crystallize human and *S*. *cerevisiae* Apc4–Apc5 were unsuccessful. We also determined that the N-terminus of Apc5 (residues 1–163) (Apc5^N^) interacts with Apc4 by coexpressing Apc4 and Apc5^N^. This coexpression yielded a huge increase of protein amounts for human, *X*. *laevis*, *S*. *cerevisiae* and *S*. *pombe*. *X*. *laevis* Apc4–Apc5^N^ was crystallized, but diffraction was limited to 8 Å resolution.

After deleting the predicted unstructured C-terminal residues 742–789 of Apc4, we purified the *X*. *laevis* Apc4–Apc5^N^ complex and we isolated Apc4 and Apc5^N^ proteins from a single purification. All of these crystallized. Apc4 and Apc5^N^ crystals were finally optimized for structure determination (see below). The *X*. *laevis* Apc4 crystal structure was used as a search object to determine the full-length human Apc4 crystal structure by molecular replacement.

#### Cloning, expression, purification and crystallization

To clone *X*. *laevis* Apc4 and the Apc5 N-terminal domain, we amplified and modified Apc4^1 -741^ and Apc5^1 -163,E163D^ (Apc5^N^) gene fragments by PCR from *X*. *laevis* Apc4 and Apc5 cDNA clones and cloned them into pU1 plasmid as previously described [54]. A double StrepII tag together with a TEV cleavage site was attached to the C-terminus of Apc5^N^. The proteins were expressed in the insect cell baculovirus system. Protein expression with selenomethionine labeling was performed in High 5 cells by a protocol modified from Ref. [57]. In brief, 16 h post-infection, cells from a 3.5-L culture were spun at 300*g* for 15 min at 22 °C, the supernatant was discarded and the cells was resuspended in 2.5 L of cysteine- and methionine-free Sf900II SFM media with 150 mg/L of l-cysteine. After starving the cells for 6 h, the media was replaced with 3.5 L of cysteine- and methionine-free Sf900II SFM media with 150 mg/L of l-cysteine and 250 mg/L of l-selenomethionine (Molecular Dimensions MD12-503). The cells were incubated under the same conditions and were harvested for protein purification 18 h post-selenomethionine addition.

*X*. *laevis* Apc4^1 -741^ (now referred to as *X*. *laevis* Apc4) was purified by collecting the late fractions of the Strep-Tactin (Qiagen) wash step and was further purified by anion-exchange chromatography Mono Q (GE Healthcare) and Superdex 200 size-exclusion chromatography (GE Healthcare). Selenomethionine-labeled Apc4 was purified as for the native protein except for the addition of 10 mM DTT and 5 mM ethylenediaminetetraacetic acid (EDTA) in the buffer. The selenomethionine labeling was confirmed by mass spectrometry. *Xenopus* Apc5^N^ was purified using a combination of Strep-Tactin, TEV cleavage, Mono Q anion-exchange chromatography, a Ni-NTA column to trap TEV and Superdex 75 size-exclusion chromatography.

To crystallize *X*. *laevis* Apc4, we concentrated the protein to 3.5 mg/mL in a buffer of 20 mM Hepes (pH 8.0), 200 mM NaCl and 2 mM DTT. Initial crystals were obtained by vapor diffusion in sitting drop in a buffer containing 0.1 M sodium citrate 5.0, 8% (w/v) polyethylene glycol (PEG) 8000. By seeding with the initial crystals, we grew large crystals in a buffer containing 0.1 M sodium citrate 5.0, 3% (w/v) PEG 8000, 250 mM magnesium acetate, 10 mM Tris–HCl (pH 8.5), 200 mM NDSB 211, 4% (v/v) ethylene glycol and 2 mM DTT. Crystals were cryoprotected in the same buffer with addition of 30% (v/v) glycerol prior to freezing in liquid nitrogen.

To crystallize *X*. *laevis* Apc5^N^, we concentrated the protein to 3.4 mg/mL. Plate-like crystals were grown in a buffer containing 40 mM sodium propionate, 20 mM sodium cacodylate, 40 mM 2-[bis(2-hydroxyethyl)amino]-2-(hydroxymethyl)propane-1,3-diol (Bistris) propane (pH 7.0) and 25% (v/v) PEG 1500. Larger crystals were obtained by seeding. For heavy metal labeling, we soaked crystals with 10 mM KAu(CN)_2_ or 1 mM ethylmercury *p*-toluene sulfonamide in a buffer containing 20 mM Hepes, 200 mM NaCl, 40 mM sodium propionate, 20 mM sodium cacodylate, 40 mM Bistris propane (pH 7.0) and 25% (v/v) PEG 1500 for 1 h before being harvested in the same buffer with addition of 15% (v/v) glycerol.

Human Apc4 was purified as a bi-product from the APC/C expression system [54]. The protein was purified using a combination of Strep-Tactin, Mono Q anion-exchange chromatography and Superdex 200 size-exclusion chromatography. The protein was concentrated to 4 mg/mL in a buffer containing 20 mM Hepes (pH 8.0), 150 mM NaCl and 1 mM DTT. Initial crystals were obtained by vapor diffusion in a buffer containing 20 mM sodium propionate, 10 mM sodium cacodylate, 20 mM Bistris propane (pH 9.0) and 25% (v/v) PEG 1500. Seeding with the initial crystals, we grew human Apc4 crystals by vapor diffusion in a buffer containing 10 mM malic acid, 20 mM Mes, 20 mM Tris–HCl (pH 8.0), 160 mM NDSB 196, 10 mM EDTA and 19% (v/v) PEG 1500. Crystals were incubated in a cryoprotection buffer comprising 10 mM malic acid, 20 mM Mes, 20 mM Tris–HCl (pH 8.0), 160 mM NDSB 196, 10 mM EDTA, 22% (v/v) PEG 1500 and 20% (v/v) ethylene glycol prior to freezing in liquid nitrogen.

### Data collection and processing

#### *X*. *laevis* Apc5^N^

The diffraction data for the native crystal were collected to a resolution of 2.2 Å on I04 beamline at Diamond Light Source. Derivative data were collected on beamline I24 for a crystal soaked in 10 mM KAu(CN)_2_ for 30 min at the gold L-III edge energy of 12.031 keV and a crystal soaked in 1 mM ethylmercury *p*-toluene sulfonamide for 30 min at the mercury L-III edge energy of 12.299 keV to a resolution of 2.4 Å. Data were processed and scaled using Xds [58] and the CCP4 program Aimless/TRUNCATE [59], respectively. The derivatives were scaled to the native and the mean empirical ratios between the anomalous and isomorphous differences, and *K*_emp_ [(F^+^_PH_ − F^−^_PH_)/F_PH_ − F_P_] was 5.65 for the gold and was 5.56 for the mercury data.

#### *X*. *laevis* Apc4

Several native and heavy-atom derivative datasets were collected and phasing attempts yielded reasonable phases. However, selenomethionine derivative data gave optimum phases with interpretable maps and were finally used to determine the structure. Data were collected on the Diamond Light Source I24 beamline using the defocused beamsize of 20 μm × 20 μm. The crystal morphology of long needles facilitated a line-scan data collection strategy. Data were collected on the Se-edge peak energy of 12,663 eV and three datasets from two crystals were combined generating a 3.8-Å-resolution peak dataset. On a later visit to I24, a high-energy remote dataset was collected on a selenomethionine derivative crystal to 3.2 Å resolution. These two datasets were scaled using SCALEIT [59] and the mean empirical ratio between the anomalous and dispersive differences, *K*_emp_ [(F^+^_PH_ − F^−^_PH_)/F_PH_ − F_P_] was 4.0 for the peak and high remote datasets.

### Human Apc4

Needle-like tiny crystals were found to be extremely radiation sensitive. Hence, diffraction data were collected from a large number of crystals on the microfocus beamline I24 of Diamond Light Source. With the inspection of *R*_merge_ and *I*/σ(*I*) of all the collected data, datasets from four crystals were indexed, merged and scaled with Xds [58] and SCALEIT [59].

### Structure determination and refinement

#### Apc5^N^

Heavy-atom detection, refinement and phasing were carried out using MIRAS with two derivatives in autoSHARP [60]. The positions and identities of three sites for each derivative along with the observed anomalous differences, isomorphous differences and structure factors were input for heavy-atom parameter refinement and phasing to autoSHARP. The data were subjected to 28 cycles of solvent flipping with the program SOLOMON. Automatic building was carried out using ARP/wARP giving an initial model with *R*_work_ of 0.27 (*R*_free_ = 0.34) after a cycle of REFMAC [61]. TLS refinement and rebuilding were carried out using REFMAC [61] and Coot [62] and to yield a model with *R*_work_/*R*_free_ of 0.20/0.24.

#### *X*. *laevis* Apc4

Heavy-atom detection, refinement and phasing were carried out using a two-wavelength anomalous diffraction strategy in autoSHARP [60]. The positions of 14 selenomethionine sites were determined, and together with the anomalous differences, the dispersive differences and structure factors were input to SHARP for heavy-atom site refinement and phasing. The data were subjected to 28 cycles of solvent flipping with the program SOLOMON. Several cycles of PARROT density modification that uses an MLHL-type likelihood target function and BUCCANEER that is optimized for tracing protein chains at low resolution with good phases gave an initial model with an *R*_work_ of 0.46 (*R*_free_ = 0.44). Extensive cycles of manual model building and refinement with PHENIX [63] and REFMAC [61] were performed.

#### Human Apc4

Phases were obtained from molecular replacement using *X*. *laevis* Apc4 as a search model, employing PHASER [64]. Electron density maps obtained from solvent flattening, using DM [59], were used for initial model building. Iterative cycles of manual model building and refinements were performed using Coot [62] and PHENIX [63], respectively. To check for model bias, we systematically calculated simulated annealing composite omit maps at various stages of model building and refinement. The refined structure was validated with MolProbity [65].

### Structure analysis

Evolutionary-conserved surfaces were determined using ConSurf [66]. Figures were generated using PyMOL‡. Regions of disorder in Apc4 and Apc5 were predicted using OnD-CRF§ [45]. TPR predictions were performed using TPRpred|| and HHrep¶ [51,52]. Protein structure predictions were performed using Phyre2 protein recognition server^a^ [53].

#### SAXS data collection and data analysis

Apc4 was concentrated to approximately 20 mg/mL. SAXS data were collected at the SWING beamline (Synchrotron Soleil, Paris, France). The beamsize was 400 μm × 100 μm, the beam energy was 12.4 keV and the flux was approximately 10^12^ photons per second. The images were collected using the AVIEV170170 CCD detector and the SAXS cell-to-detector distance was 1892 cm. The samples were loaded in gel-filtration buffer using an online HPLC device and a Shodex_KW402.5-4F size-exclusion column. The column and flow cell were maintained at 10 °C and the sample holder prior to injection was at 4 °C. Data were processed using the beamline software FOXTROT and Guinier analysis was performed using PRIMUS [48]. Theoretical SAXS profiles for Apc4 were calculated and fitted to the SAXS experimental profiles with the FoXS Web server [67]. Radius of gyration (*R*_g_) were determined from the experimental SAXS profile using GNOM [50] and calculated *R*_g_ using FoXS.

X-ray crystallographic maps and EM density maps were displayed and analyzed using Coot [62] and PyMOL. Atomic models were docked into the APC/C^Cdh1.Emi1^ cryo-EM map [44] using Chimera [46]. Local-resolution EM maps were calculated using ResMap [68].

### Accession numbers

Coordinates and structure factors have been deposited in the Protein Data Bank with accession numbers 5BPW, 5BPT and 5BPZ for human and *Xenopus* Apc4 and *Xenopus* Apc5^N^, respectively.

## Figures and Tables

**Table 1:** DALI matches (top 3 matches)

Search protein	Protein match	PDB	*Z*	RMSD (Å)	*N*_ali_	*N*_res_	% ID
Apc5^N^	RNP assembly factor	3zv0-B	5.7	3.5	115	329	10
Polybromo-1	3iu6-A	4.5	3.1	82	143	7
RBCX protein	3q20-A	4.5	3.4	77	114	10
Apc5^TPR^	Malt regulatory protein	1hz4-A	22.7	3.4	325	366	12
NPRR	4gpk-L	18.2	5.8	320	346	9
Cut9 (Apc6) (8th)	2xpi-A	16.1	3.7	340	520	12
Apc4^WD40^	Polycomb protein EED WD40 protein	3iic-A	25.0	3.1	312	357	10
eiF3 subunit WD40 protein	3zwl-D	24.2	3.1	285	352	10
WDR5 WD40 protein	2h14-A	24.0	3.1	280	303	12
Apc4^HBD^	Cytochrome *c* oxidase	1m56-C	12.0	3.0	129	265	4
Outer surface protein C	1f1m-C	10.7	2.7	133	162	11
α-Catenin	1h6g-A	10.7	2.7	129	255	6

**Table 2:** RMSD of Apc4 and Apc5 X-ray and EM models

Subunit	*H.s.* Apc4^EM^	*H.s.* Apc4^X-ray^	*X.l.* Apc4^X-ray^	*X.l.* Apc5^X-ray^
*H.s.* Apc4^EM^	—	2.4 Å	2.5 Å	—
*H.s.* Apc4^X-ray^	—	—	2.1 Å	—
*H.s.* Apc5^EM^	—	—	—	2.0 Å

**Table 3:** Intersubunit solvent-accessible surface area (Å^2^)

Subunit	Apc4	Apc5	Apc1	Apc2	Apc8	Apc15
Apc4	—	6226	0	714	0	0
Apc5	—	—	8744	0	3772	3446

Data taken from Ref. [44].

**Table 4:** Table comparing regions of disordering in APC/C subunits as observed from X-ray structures and the cryo-EM structure of APC/C^Cdh1.Emi1^

Subunit	X-ray structure				EM structure. PDB code 4UI9. *H.s.* APC/C. Ref. [44]	Disorder prediction	*H.s.* amino acids (*N*)
—	PDB code	Ref.	Construct	Disordered regions within crystal	Disordered regions	—	—
*H.s.* Apc3	4RG6	[37]	1–181, 454–830 (830)	1–4, 172–181, 768–830	171–450, 767–830 (A)^b^ 171–450, 782–830 (B)	1–7, 94–109, 172–453, 770–830	830
*H.s.* Apc4	5BPW	This study	1–808 (808)^a^	1–5, 304–317, 431–444, 458–472, 479–521, 758–808	429–438, 458–469, 758–808	1–9, 125–164, 557–570, 753–808	808
*X.l.* Apc5	5BPZ	This study	1–163 (759)	1–26	1–26, 170–205	1–8, 166–212, 284–298, 741–755	755
*H.s.* Apc6	3YUM	[33]	212–539 (620)	212–228, 530–539	96–126, 528–620 (A) 96–123, 534–620 (B)	1–12, 110–130, 546–620	620
*S.p.* Apc6	2XPI	[35]	1–597 (671)	1–44, 61–80, 596–597	As above	As above	As above
*H.s.* Apc7	3FFL	[56]	1–147 (599)	1–20, 77–96, 147	1–35, 111–131, 553–599 (A) 1–35, 111–131, 541–599 (B)	1–8, 60–71, 98–140, 534–599	599
*S.p.* Apc8	3ZN3	[34]	19–302 (565)	57–77, 136–151, 285–292	1–25, 501–508, 558–597 (A) 1–25, 501–508, 558–597 (B)	1–10, 125–167, 571–597	597
*H.s.* Apc10	1JHJ	[70]	1–185 (185)	163–185	None	1–28, 180–185	185
*H.s.* Apc11	4R2Y	[31]	17–84 (84)	17–20	None	None	84
*H.s.* Apc12	3YUM	[33]	1–85 (85)	27–85	26–85	1–9, 25–85	85
*S.p.* Apc12	2XPI	[35]	1–80 (80)	25–80	As above	As above	As above
*H.s.* Apc16	4RG6	[37]	74–110 (110)	108–110	1–51	1–53, 94–110	110
*S.c.* Cdh1	4BH6	[39]	241–550 (566)	1–4, 549–550	1–41, 68–87, 109–124, 133–145, 164–168, 471–482	1–93, 131–146, 152–172, 490–496	496

a Numbers in parentheses indicate protein residue number.

b Letters in parentheses indicate subunit label.

## Appendix A Supplementary data

Supplementary Fig. 1. Multiple sequence alignment of Apc4. This figure was generated using ALSCRIPT [69]. Invariant residues are shown as white on red, and conserved residues are shown as black on yellow. Secondary structural elements as observed in the human APC/C^Cdh1.Emi1^ EM density map are indicated and labeled. Helices that are disordered in the crystal structures are colored gray. Gray broken lines indicate disordered residues of Apc4 in the APC/C^Cdh1.Emi1^ cryo-EM structure. *S.c.*: *S*. *cerevisiae*; *S.p.*: *S*. *pombe*; *A.t.*: *Arabidopsis thaliana*; *D.m.*: *Drosophila melanogaster*; *H.s.*: *Homo sapiens*; *X.l.*: *X*. *laevis*. Supplementary Fig. 2. (a) Fit of Apc4 and Apc5 atomic models to the cryo-EM map (in blue mesh) of human APC/C^Cdh1.Emi1^[44]. (b) Apc4 and Apc5 are located within the platform region of the APC/C. Cartoon of the APC/C^Cdh1.Emi1^ model with Apc4 and Apc5 colored. Atomic model from Ref. [44] (PDB code 4ui9). (c) SAXS profile of human Apc4. The experimental SAXS profile shows a good fit to the computed SAXS profile of the Apc4 model (χ^2^ = 5.97). The experimental radius of gyration (*R*_g_) for Apc4 is 34.9 Å and the calculated radius of gyration the EM-refined Apc4 coordinates is 34.8 Å. Supplementary Fig. 3. Multiple sequence alignment of Apc5. This figure was generated using ALSCRIPT [69]. Invariant residues are shown as white on red, and conserved residues are shown as black on yellow. Secondary structural elements as observed in the APC/C^Cdh1.Emi1^ EM density map are indicated and labeled. Gray broken lines indicate disordered residues of Apc5 in the APC/C^Cdh1.Emi1^ cryo-EM structure. *S.c.*: *S*. *cerevisiae*; *S.p.*: *S*. *pombe*; *D.m.*: *D*. *melanogaster*; *X.t.*: *Xenopus tropicalis*; *C.e.*: *Caenorhabditis elegans*; *M.m.*: *Mus musculus*; *H.s.*: *H*. *sapiens*. Supplementary Fig. 4. Comparison of EM density maps of APC/C^Cdh1.Emi1^ and crystal structure 2*F*_o_ − *F*_c_ maps of Apc5. (a) Stereoview of the EM density map and Apc5 coordinates. Main chain is shown as a cartoon, and amino acid side chains are shown as sticks. (b) Stereoview of the 2*F*_o_ − *F*_c_ density map contoured at 1σ and Apc5^N^ coordinates. Main chain is shown as a cartoon, and amino acid side chains are shown as sticks. Supplementary Fig. 5. Apc5^TPR^ shares close structural similarity to other TPR superhelices. (a) Stereoview showing a superimposition of Apc5^TPR^ (blue) onto MALT (salmon). (b). Stereoview showing a superimposition of Apc5^TPR^ (blue) onto *S*. *pombe* Apc6 (salmon). EM coordinates of human Apc5 are shown. Supplementary Fig. 6. Local-resolution map of the APC/C^Cdh1.Emi1^ cryo-EM structure. (a) Molecular surface of APC/C^Cdh1.Emi1^ EM density map color-coded according to local resolution. Calculated using ResMap [68]. (b). Molecular surface of APC/C^Cdh1.Emi1^ EM density map color-coding according to subunit assignments. Table S1. *X*. *laevis* Apc4 data collection and refinement statistics Table S2. Human Apc4 data collection and refinement statistics Table S3 *X*. *laevis* Apc5N data collection and refinement statistics

## Footnotes

‡ pymol.org.

§ http://babel.ucmp.umu.se/ond-crf/.

|| http://toolkit.tuebingen.mpg.de/tprpred.

¶ http://toolkit.tuebingen.mpg.de/HHrep.

a http://www.sbg.bio.ic.ac.uk/phyre2/html/page.cgi?id=index.
