# Supplementary material for: Atomic-Resolution Structures of the APC/C Subunits Apc4 and the Apc5 N-Terminal Domain
Source: J Mol Biol. 2015 Oct 9;427(20):3300–15. doi: 10.1016/j.jmb.2015.08.023 (PMC4590430; doi:10.1016/j.jmb.2015.08.023)
Supplement: Supplementary file 1 — Supplementary Fig. 1. Multiple sequence alignment of Apc4. This figure was generated using ALSCRIPT [69]. Invariant residues are shown as white on red, and conserved residues are shown as black on yellow. Secondary structural elements as observed in the human APC/CCdh1.Emi1 EM density map are indicated and labeled. Helices that are disordered in the crystal structures are colored gray. Gray broken lines indicate disordered residues of Apc4 in the APC/CCdh1.Emi1 cryo-EM structure. S.c.: S. cerevisiae; S.p.: S. pombe; A.t.: Arabidopsis thaliana; D.m.: Drosophila melanogaster; H.s.: Homo sapiens; X.l.: X. laevis. Supplementary Fig. 2. (a) Fit of Apc4 and Apc5 atomic models to the cryo-EM map (in blue mesh) of human APC/CCdh1.Emi1[44]. (b) Apc4 and Apc5 are located within the platform region of the APC/C. Cartoon of the APC/CCdh1.Emi1 model with Apc4 and Apc5 colored. Atomic model from Ref. [44] (PDB code 4ui9). (c) SAXS profile of human Apc4. The experimental SAXS profile shows a good fit to the computed SAXS profile of the Apc4 model (χ2 = 5.97). The experimental radius of gyration (Rg) for Apc4 is 34.9 Å and the calculated radius of gyration the EM-refined Apc4 coordinates is 34.8 Å. Supplementary Fig. 3. Multiple sequence alignment of Apc5. This figure was generated using ALSCRIPT [69]. Invariant residues are shown as white on red, and conserved residues are shown as black on yellow. Secondary structural elements as observed in the APC/CCdh1.Emi1 EM density map are indicated and labeled. Gray broken lines indicate disordered residues of Apc5 in the APC/CCdh1.Emi1 cryo-EM structure. S.c.: S. cerevisiae; S.p.: S. pombe; D.m.: D. melanogaster; X.t.: Xenopus tropicalis; C.e.: Caenorhabditis elegans; M.m.: Mus musculus; H.s.: H. sapiens. Supplementary Fig. 4. Comparison of EM density maps of APC/CCdh1.Emi1 and crystal structure 2Fo − Fc maps of Apc5. (a) Stereoview of the EM density map and Apc5 coordinates. Main chain is shown as a cartoon, and amino acid side chains ar [file mmc1.pdf]

Supplementary Figure 1

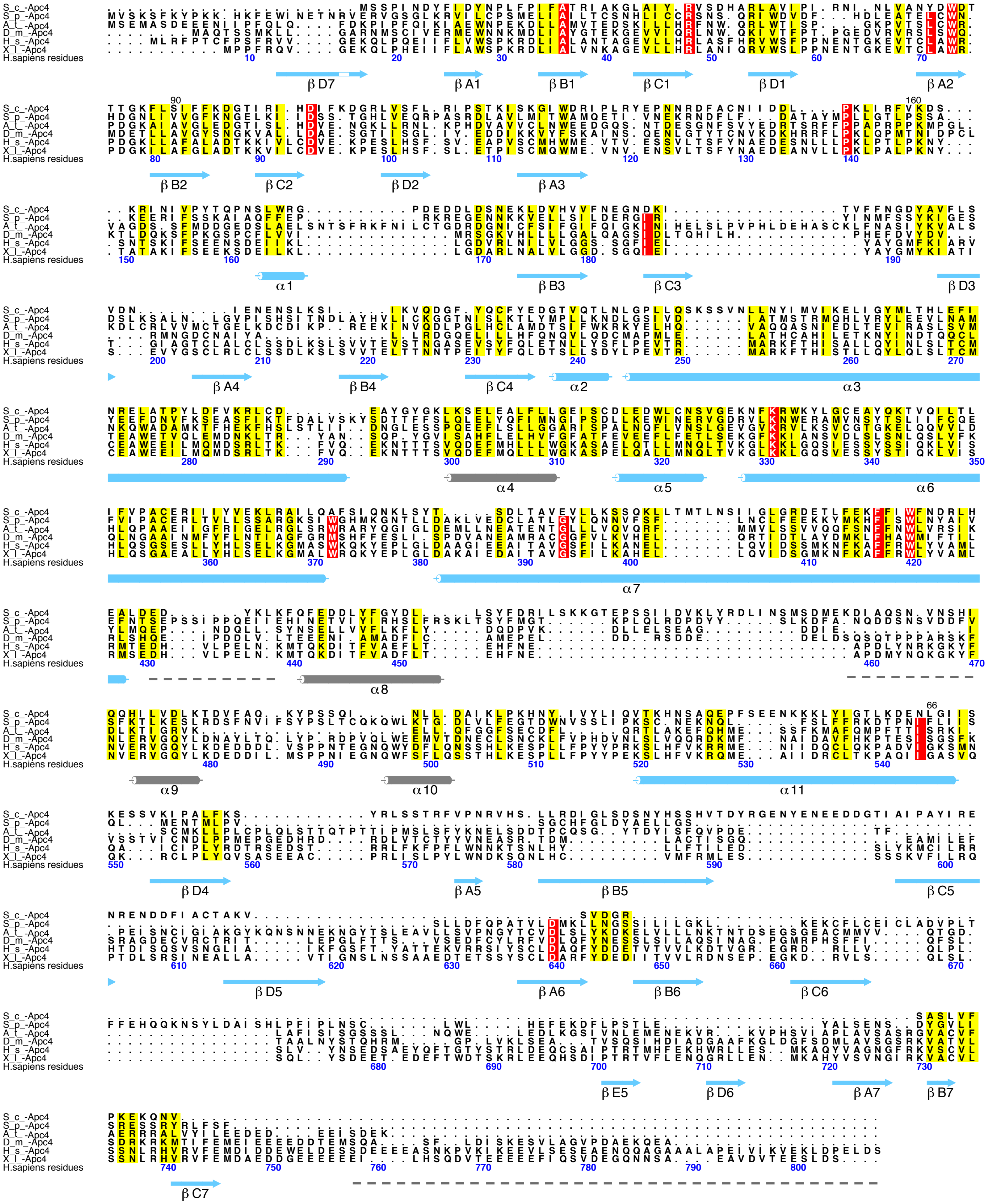

Supplementary Figure 2

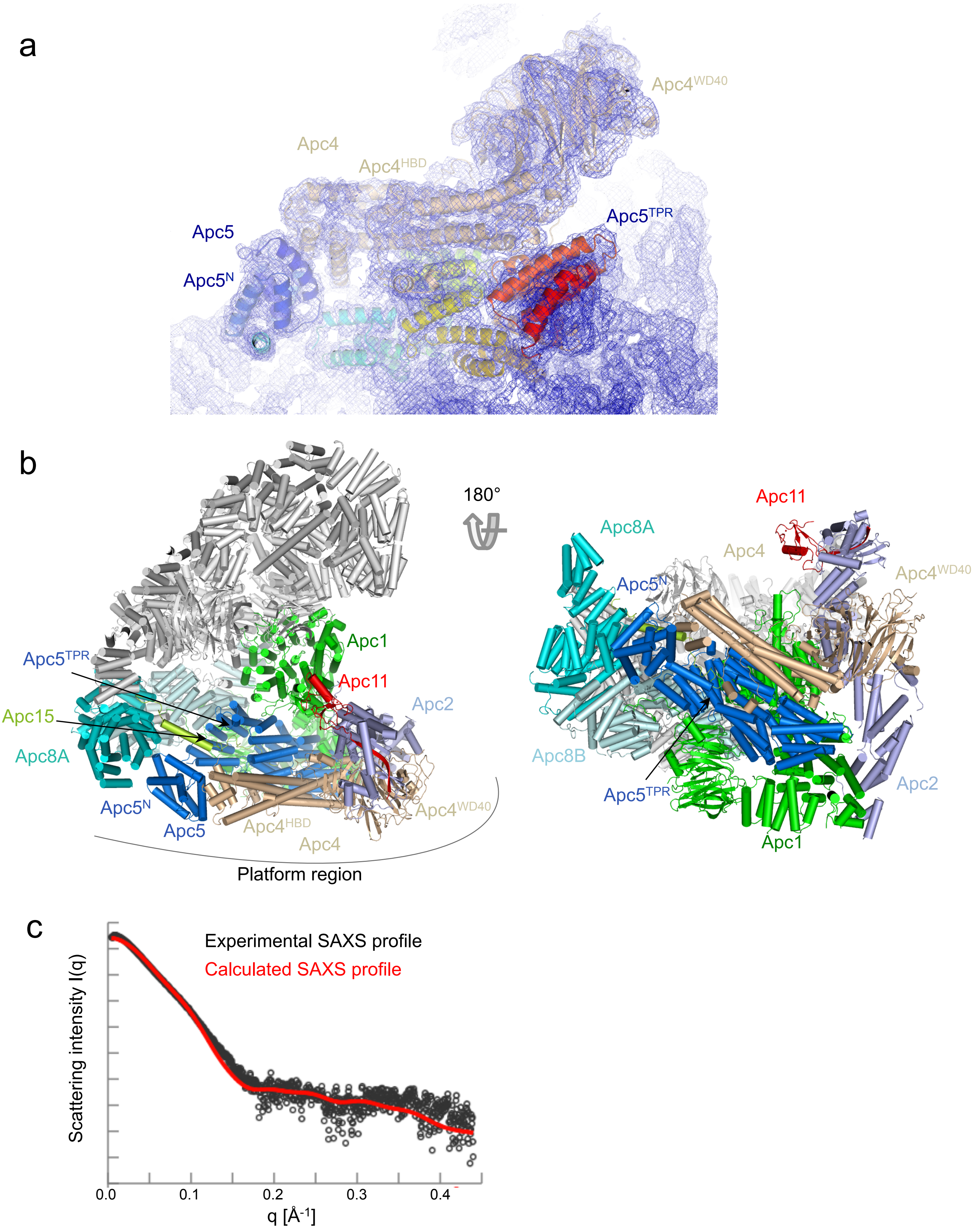

Supplementary Figure 3

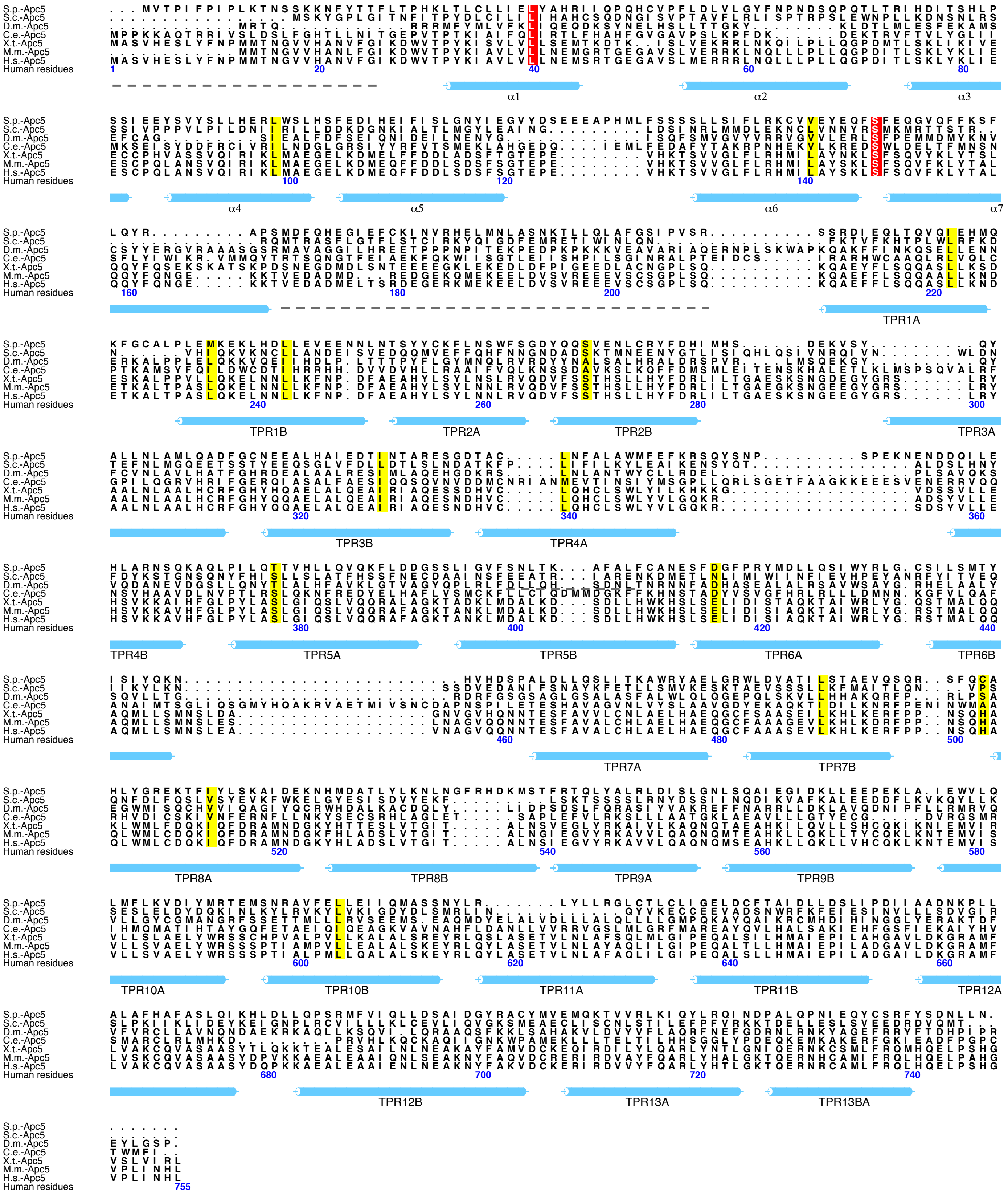

Supplementary Figure 4

a

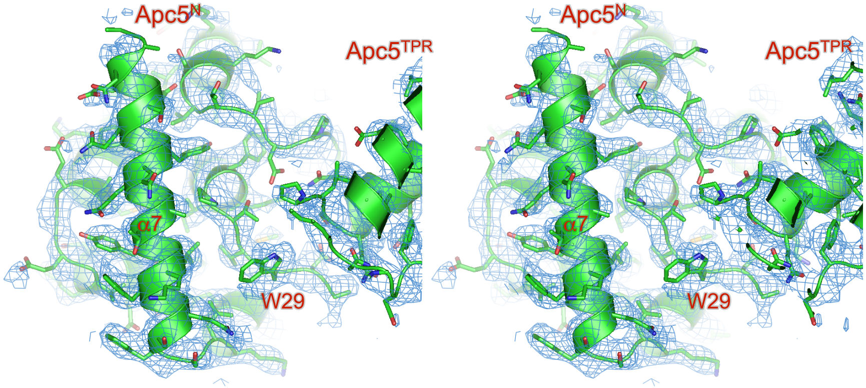

b

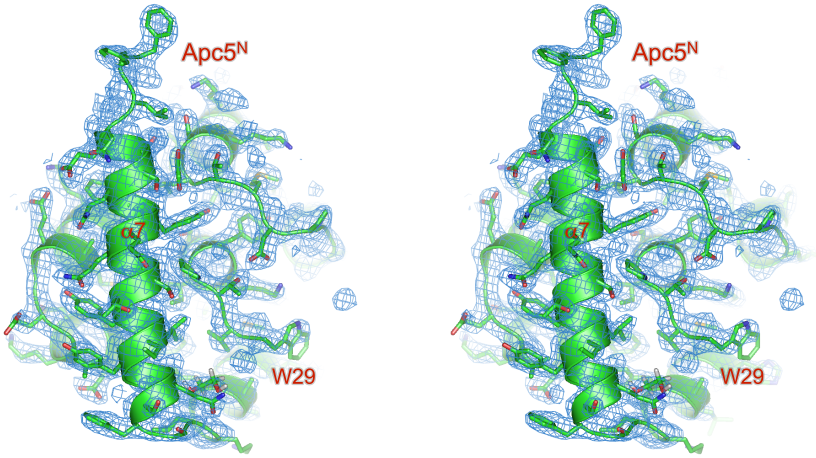

Supplementary Figure 5

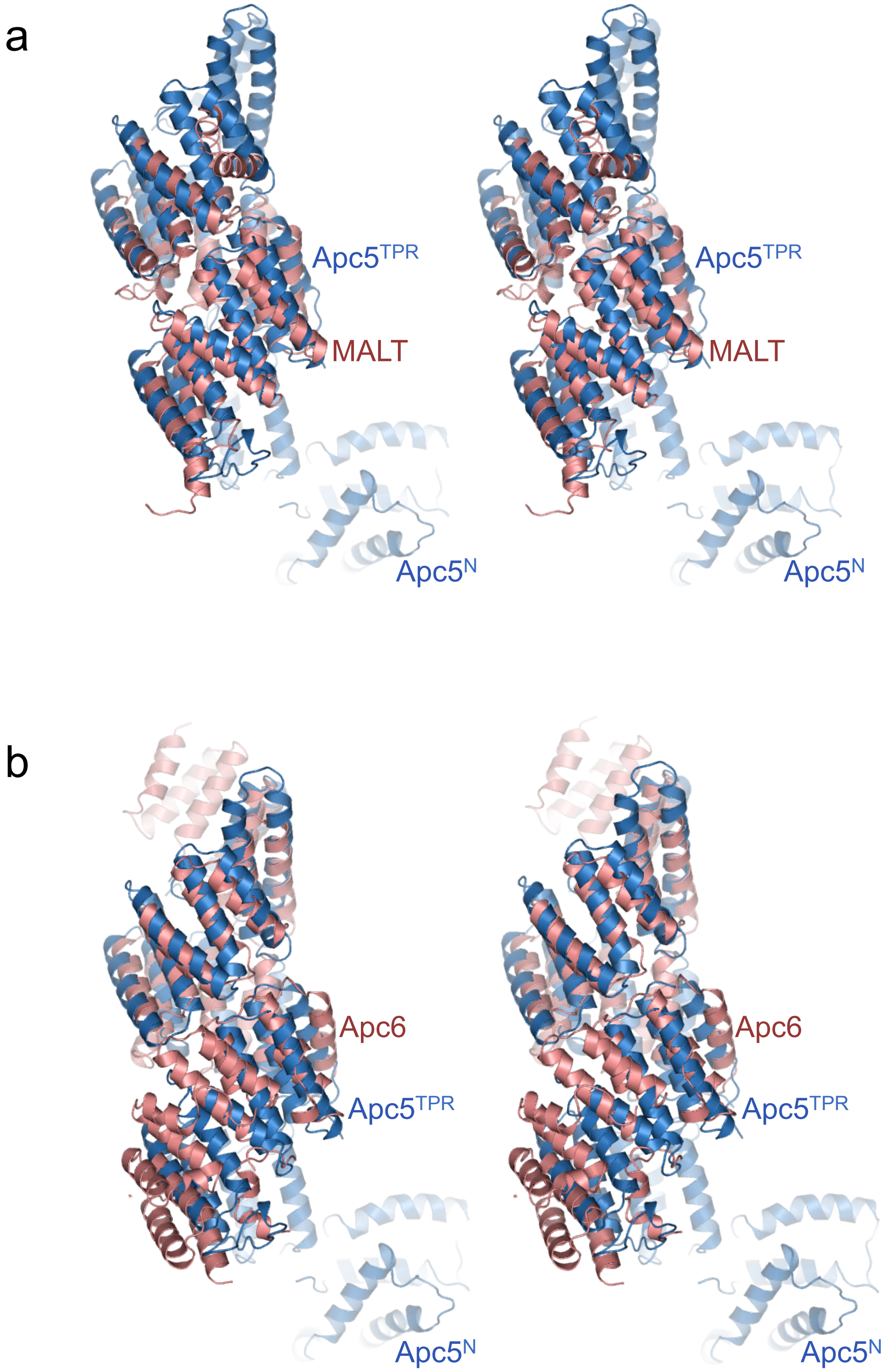

Supplementary Figure 6

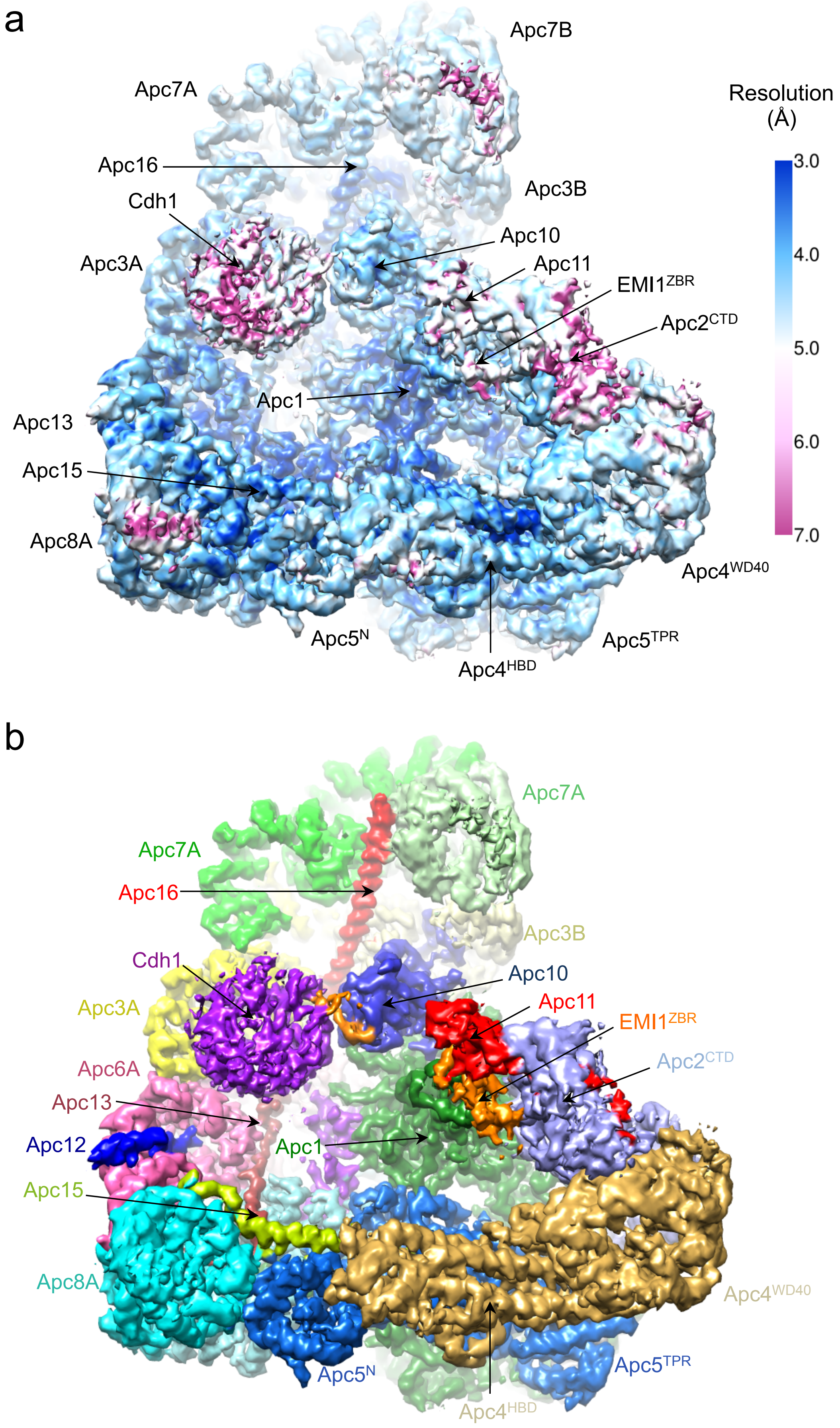

Table S1. ***Xenopus laevis*** **Apc4 Data Collection and Refinement Statistics**

**Data Collection Statistics**	**SeMet**	**SeMet**
Beam line	DLS I24	DLS I24
No. of Crystals used	2	1
Space Group	$P4_1$	$P4_1$
**a** (Å)	90.67	90.47
**b** (Å)	90.67	90.47
**c** (Å)	116.15	115.59
Wavelength (**Å**)	0.979	0.969
Solvent fraction	0.537	0.537
Resolution limits (Å)<br>(Outer resolution shell range Å)	3.8<br>(8.5 – 3.8)	3.2<br>(3.42 - 3.2)
R_{merge}	0.206 (0.631)	0.20 (0.82)
I/σ (I)	21.0 (9.7)	13.3 (1.6)
Total number unique	9360 (2635)	15477 (2759)
Completeness (%)	99.9 (99.9)	99.8 (98.5)
Multiplicity	27.7 (27.7)	7.5 (7.7)
Anomalous completeness (%)	100.0 (99.9)	99.5 (98.5)
Anomalous multiplicity	13.8 (13.6)	3.8 (3.7)
**Refinement Statistics**		
Resolution limits (Å)	-	29.25 - 3.2
Working set	-	14398
Test set	-	1016
R_{work}/R_{free}	-	0.222/0.265
No. of Protein atoms (N)	-	4786
Protein atoms ($Å^2$)	-	92.38
Bond length (Å)	-	0.010
Bond angles (°)	-	1.34
Ramchandran Plot Statistics		
Preferred (%)	-	93.1
Allowed (%)	-	3.6

Table S2. Human Apc4 Data Collection and Refinement Statistics

**Data Collection Statistics**	**Native**
Beam line	DLS I24
No. of Crystals used	4
Space Group	$P42_12$
**a** (Å)	139.2
**b** (Å)	139.2
**c** (Å)	156.1
**β(°)**	90
**γ(°)**	90
Z	1
Solvent fraction	0.7
Resolution limits (Å)<br>(Outer resolution shell range Å)	57.8 - 3.40<br>(3.67 - 3.40)
R_{merge}	0.182 (0.60)
I/σ (I)	5.16 (2.3)
Total number unique	21671 (4371)
Completeness (%)	99.8 (99.6)
Multiplicity	6.5 (6.5)
**Refinement Statistics**	
Resolution limits (Å)	51.9 - 3.4
Working set (N)	20931
Test set (N)	700
R_{work}/R_{free}	0.225/0.281
Protein atoms (N)	5087
Protein atoms (Å^2)	88.90
Bond length (Å)	0.01
Bond angles (°)	1.65
Ramchandran Plot Statistics	
Preferred (%)	94.71
Allowed (%)	5.29

Table S3 *Xenopus laevis* Apc5^N Data Collection and Refinement Statistics

**Data Collection Statistics**	**Native**	**$KAu(CN)_2$**	**EMTS**
Beam line	DLS I04	DLS I24	DLS I24
No. of Crystals used	1	1	1
Space Group	$P2_12_12$	$P2_12_12$	$P2_12_12$
**a** (Å)	40.97	40.97	40.98
**b** (Å)	57.30	56.57	57.75
**c** (Å)	63.62	63.85	63.78
Wavelength (**Å**)	0.9795	1.0392	1.0081
Solvent fraction	0.307	0.307	0.307
Resolution limits **(**Å)<br>(Outer resolution shell range Å)	63.62 - 2.18<br>(2.24 - 2.18)	40.97 - 2.46	42.81 - 2.41
R_{merge}	0.055 (0.663)	0.078 (0.596)	0.093 (0.794)
I/σ (I)	28.5 (4.5)	17.3 (2.6)	18.7 (3.4)
Total number unique	8213 (590)	5666 (350)	6244 (450)
Completeness (%)	99.4 (99.1)	98.4 (87.7)	99.9 (100.0)
Multiplicity	12.8 (12.8)	9.4 (5.9)	12.7 (13.2)
Anomalous completeness (%)	-	98.3 (87.4)	99.9 (100.0)
Anomalous multiplicity	-	5.1 (2.9)	7.0 (7.0)
**Refinement Statistics**			
Resolution limits **(**Å)	63.62 – 2.18	-	-
Working set	8007	-	-
Test set	395	-	-
R_{work}/R_{free}	0.211/0.226	-	-
Protein atoms (N)	1138	-	-
Solvent atoms (N)	48	-	-
Mean B-factors		-	-
Protein atoms (**Å**^{2})	42.90	-	-
Bond length (**Å**)	0.0083	-	-
Bond angles (°)	0.96	-	-
Ramchandran Plot Statistics			
Preferred (%)	94.93	-	-
Allowed (%)	2.90	-	-
MolProbity score	1.65	-	-

Apc5 residues 47-54 α1/α2 loop are disordered (cryo-EM map) 118-127, α5/α6 loop are disordered (X-ray).
